# Early recurrence of ruptured distal anterior cerebral artery aneurysm following stent-assisted coiling and salvage treatment with flow diverter: a case report and mechanistic analysis

**DOI:** 10.3389/fsurg.2026.1676032

**Published:** 2026-05-20

**Authors:** Li Chen, Jia Q. Sun, Li Zhang, Yuan F. Du, Yang Cao, Jie Zhang, Wen H. Yu, Hao Wang

**Affiliations:** 1 Department of Neurosurgery, The 2nd Affiliated Hospital of Zhejiang Chinese Medical University, Hangzhou, China; 2 The Fourth School of Clinical Medicine, Zhejiang Chinese Medical University, Hangzhou, China; 3 Department of Neurosurgery, Affiliated Hangzhou First People's Hospital, School of Medicine, Westlake University, Hangzhou, China

**Keywords:** distal anterior cerebral artery, early recurrence, endovascular treatment, flow diverter, mechanistic analysis, ruptured aneurysm, stent-assisted coiling, subarachnoid hemorrhage

## Abstract

This article reports a novel case of a ruptured distal anterior cerebral artery (DACA) aneurysm. Initial laser-cut stent-assisted coiling resulted in significant recurrence at 1 month follow-up. Salvage treatment was then successfully performed with a flow diverter (FD) telescopically deployed within the original stent. One month follow-up angiography showed complete aneurysm obligation, classified as O'Kelly-Marotta (OKM) grade D, with unobstructed parent artery and branches. We also explored the possible mechanisms of recurrence and treatment strategies. To the best of our knowledge, this is the first reported case of successfully retreating a recurrent ruptured DACA aneurysm from a less than 2 mm parent artery using FD telescoping after failed stent-assisted coiling.

## Case report

**Figure 1:**
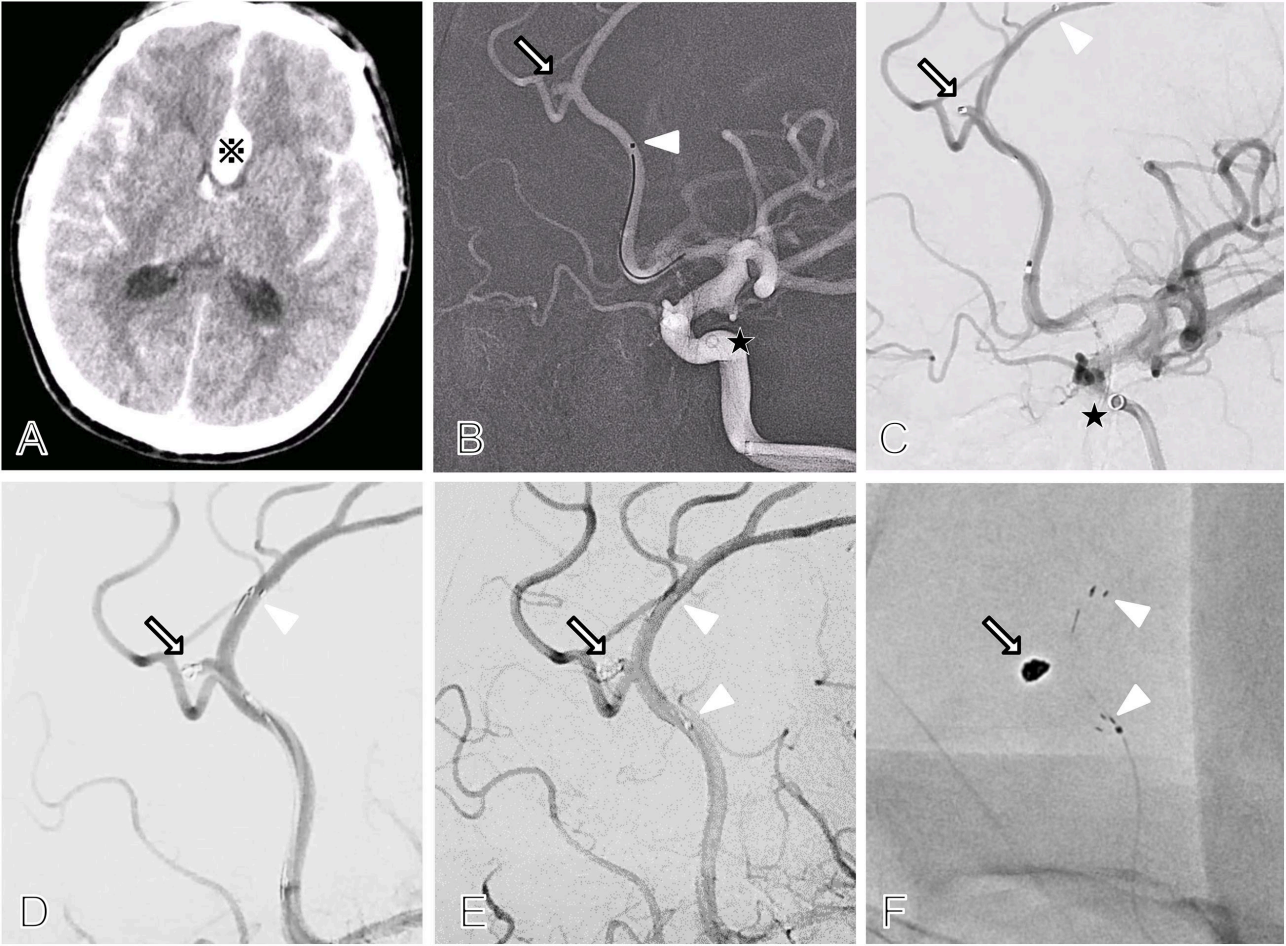
Initial imaging and Atlas^TM^ stent-assisted coiling of a ruptured distal anterior cerebral artery aneurysm. **A**, CT reveals extensive subarachnoid hemorrhage accompanied by an interhemispheric hematoma (※). **B**, Working-angle angiography shows the stent microcatheter (white arrowhead) approaching the distal anterior cerebral artery aneurysm (arrow) with support from the distal access catheter (black pentagram). **C**, The stent microcatheter (white arrowhead) is advanced beyond the aneurysm, while the embolization microcatheter enters the aneurysm (arrow). The distal access catheter tip (black pentagram) is positioned below the ophthalmic artery of the internal carotid artery, with the entire system having a long path and weak support. **D**, The distal end of the Atlas^TM^ stent (white arrowhead) is deployed. After partial stent release, the embolization microcatheter is stabilized using the jailing technique, followed by coil placement within the aneurysm (arrow). **E**, The proximal and distal ends of the Atlas^TM^ stent (white arrowheads) are fully deployed, achieving Raymond–Roy Occlusion Classification grade 2 coil packing with residual neck filling (arrow). The pericallosal and callosomarginal arteries remain patent. **F**, The mask image demonstrates the proximal and distal ends of the stent (white arrowheads) and the coil mass within the aneurysm (arrow).

A 57-year-old male admitted with sudden-onset headache and unconsciousness. Head CT revealed subarachnoid hemorrhage with interhemispheric fissure and periventricular hematoma (Figure 1A). CTA and DSA showed a ruptured aneurysm in the distal A3 segment of the right anterior cerebral artery. The patient had no significant past medical history. Emergency cerebral angiography confirmed a ruptured aneurysm at the pericallosal-callosomarginal artery bifurcation, with the two branches forming an approximately 180° angle. The aneurysm measured 2.2 mm × 1.6 mm with a 1.7 mm neck, yielding a dome-to-neck ratio of 1.3, consistent with a wide-neck aneurysm (Figure 1B).

Initial coil embolization with a Headway^TM^ 17 microcatheter failed due to an S-shaped angle between the proximal anterior cerebral artery and the aneurysm's long axis, which impeded the microcatheter navigation and caused the intermediate catheter kickback. The instability of the microcatheter made satisfactory coil framing unachievable (Figures 1B,C). Additionally, given the aneurysm's small size and relatively wide neck, the procedure was switched to stent-assisted coil embolization.

Another Headway^TM^ 17 was used to partially deploy an Atlas^TM^ 3 × 15 stent at the aneurysm's proximal neck. Under stent protection, four coils (1.5 × 3, 1 × 3  , and two 1 × 2 mm) were successfully implanted, followed by full stent was deployment. The final result achieved a Raymond–Roy class (1) II, indicating minimal residual filling at the aneurysm neck, with the parent artery and its branches remaining patent (Figures 1D–F). The patient experienced no postoperative complications.

**Figure 2:**
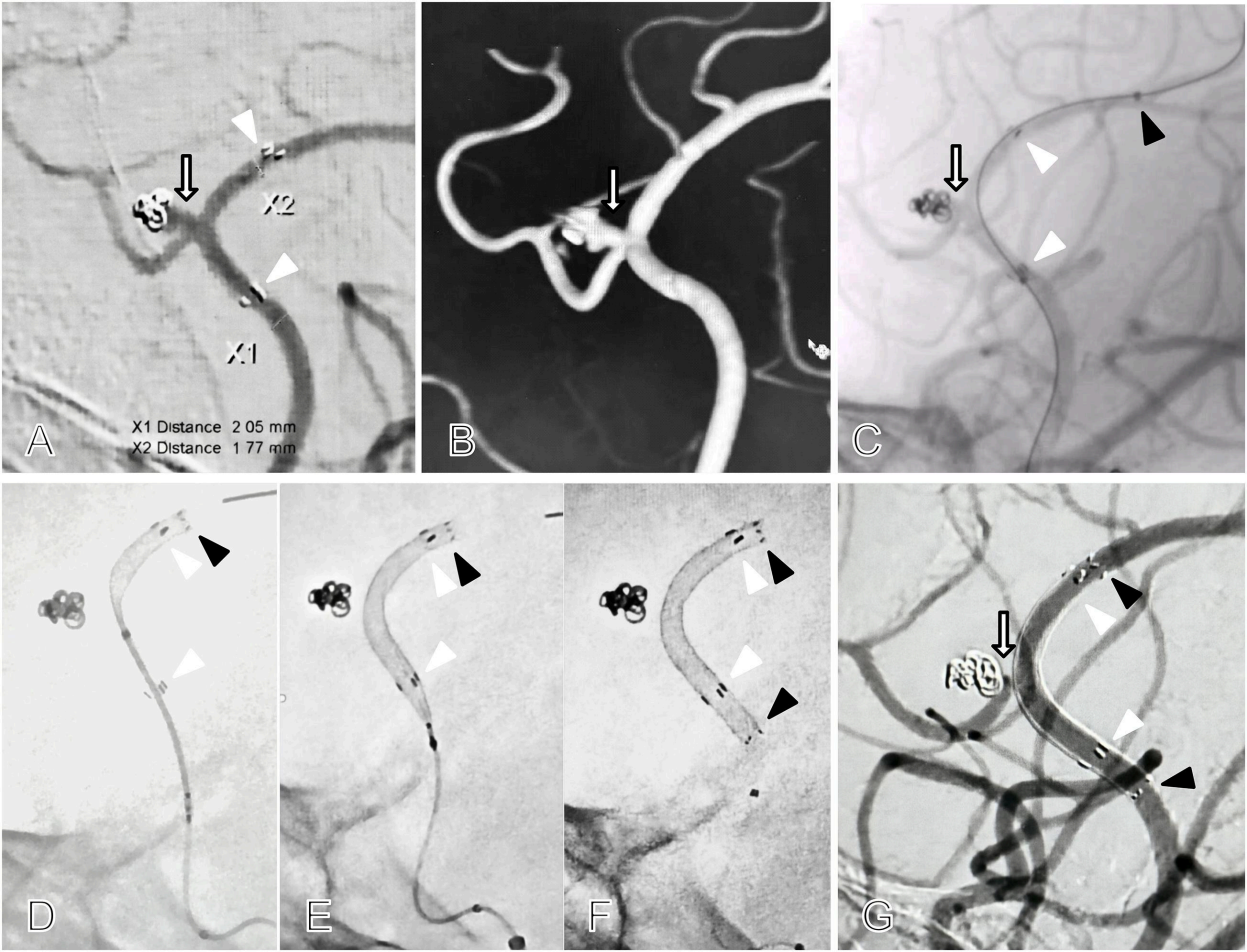
Significant recurrence at 35-day follow-up after Atlas^TM^ stent-assisted coiling of a ruptured distal anterior cerebral artery aneurysm, salvaged with a flow-diverting stent. **A** and **B**, 2D DSA and 3D DSA from the initial working-angle reveal distal migration of the coil mass, with marked recurrence at the aneurysm neck and body (arrow), exceeding the size of the initially ruptured aneurysm. The Atlas^TM^ stent remains in place, with good apposition at both proximal and distal ends (white arrowheads). The diameters of the pericallosal artery proximal and distal to the aneurysm were 2.05 mm and 1.77 mm, respectively. **C**-**G**, Salvage treatment of the recurrent anterior cerebral artery aneurysm using a Qilin^TM^ flow diverter telescoped within the existing Atlas^TM^ stent. **C**, A Headway^TM^ 21 microcatheter (black arrowhead) was carefully advanced through the existing Atlas^TM^ stent (white arrowheads) under microguidewire guidance, crossing the recurrent aneurysm neck (white arrow) to reach the A4 segment of the pericallosal artery. **D**, After adjusting system tension, the distal marker of the 2.5 mm × 20 mm flow diverter (black arrowhead) was slowly advanced approximately 3 mm beyond the distal end of the existing Atlas^TM^ stent (upper white arrowhead) and deployed *in situ* using a 1:1 push-and-pull technique. **E**, With the flow diverter's distal marker (black arrowhead) stabilized, the device was slowly deployed within the existing Atlas^TM^ stent (white arrowheads) until nearing the detachment point. **F**, The flow diverter was fully deployed, with its proximal end (lower black arrowhead) positioned just below the proximal end of the existing Atlas^TM^ stent (lower white arrowhead). **G**, One month follow-up after salvage treatment shows the flow diverter (black arrowheads) telescoped bidirectional within the Atlas^TM^ stent (white arrowheads), with complete aneurysm obliteration and preservation of the pericallosal and callosomarginal arteries (arrow).

On the 35th postoperative day, follow-up imaging revealed significant radiographic recurrence with enlargement of the original A3 segment aneurysm, which measured 4.2 mm × 2.8 mm. Coils migration to the distal sac was noted, with the residual size of the aneurysm body and neck exceeding its pre-ruptured dimensions (Figures 2A,B). In this situation, microcatheter instability and an indwelling stent precluded safe traversal of the stent mesh for additional coil packing, making clipping equally also unsuitable. Flow diversion was therefore considered the preferred strategy. Following DAPT preparation for 5 dyas, comprising aspirin 100 mg daily and clopidogrel 75 mg daily, remedial of a FD implantation was performed under general anesthesia. A 5F intermediate catheter, supported by a 6F long sheath, reached the cavernous segment of the right internal carotid artery. A Headway^TM^ 21 microcatheter, guided by a Synchro^TM^ 2 microwire, was carefully navigated past the aneurysm neck and carefully traversed the existing Atlas^TM^ stent into the A4 segment of the anterior cerebral artery (Figure 2C). After tension adjustment, a 2.5 mm × 20 mm Qilin^TM^ FD was advanced and positioned with its distal marker 3 mm beyond the distal end of the original Atlas^TM^ stent. The FD was deployed *in situ* with a 1:1 push-and-pull technique, fully releasing the distal end of the FD below the proximal end of the Atlas^TM^ (Figures 2D–F). The patient was discharged uneventfully on postoperative day 3. One month imaging follow-up showed complete aneurysm obliteration, classified as OKM grading scale (2) of Grade D, while the pericallosal and callosomarginal arteries remained patent (Figure 2G). At 9 month telephone follow-up, the patient reported no neurological deficits and had returned to work.

## Discussion

DACA aneurysms have an endovascular treatment recurrence rate of 23.4% (3), with one-third requiring reintervention after simple coil embolization, which is higher than that of anterior communicating artery aneurysms (10.8% recurrence, 3.5% retreatment rate) (4). This is attributed to the small size of DACA aneurysms and the narrow parent artery diameter (<2 mm), leading to poor microcatheter stability and insufficient embolization density (4). Recurrence rates can reach 39.1% when combined with a wide-angled (>180°) callosomarginal artery branch or irregular aneurysm morphology (3). In this case of a ruptured DACA aneurysm, initial angiography revealed all the above factors. The first treatment used the jailing technique with partial deployment of an Atlas^TM^ stent followed by coil packing, aiming to stabilize the microcatheter, reduce kickback, improve embolization density, and leverage the stent's dome effect to protect the callosomarginal artery origin. However, treatment was halted due to microcatheter retraction to the aneurysm neck and coil loops entering the callosomarginal artery, ultimately achieving a Raymond grade 2 occlusion.

One month follow-up, the DACA aneurysm showed significant recurrence and enlargement, with the coil mass displaced distally. The residual aneurysm exceeded its initial rupture size. Apart from the predisposing factors for DACA aneurysm recurrence and the open-cell design of the Atlas^TM^ stent, which has low metal coverage and limited hemodynamic influence, the rapid and marked recurrence of this aneurysm contrasts with the findings of Cho et al. (3), who reported no recurrence in seven cases of unruptured DACA aneurysm treated with stent-assisted coiling. This discrepancy indicates a correlation between rupture characteristics of the aneurysm and the occurrence of extensive subarachnoid hemorrhage, especially the interhemispheric hematoma.

**Figure 3:**
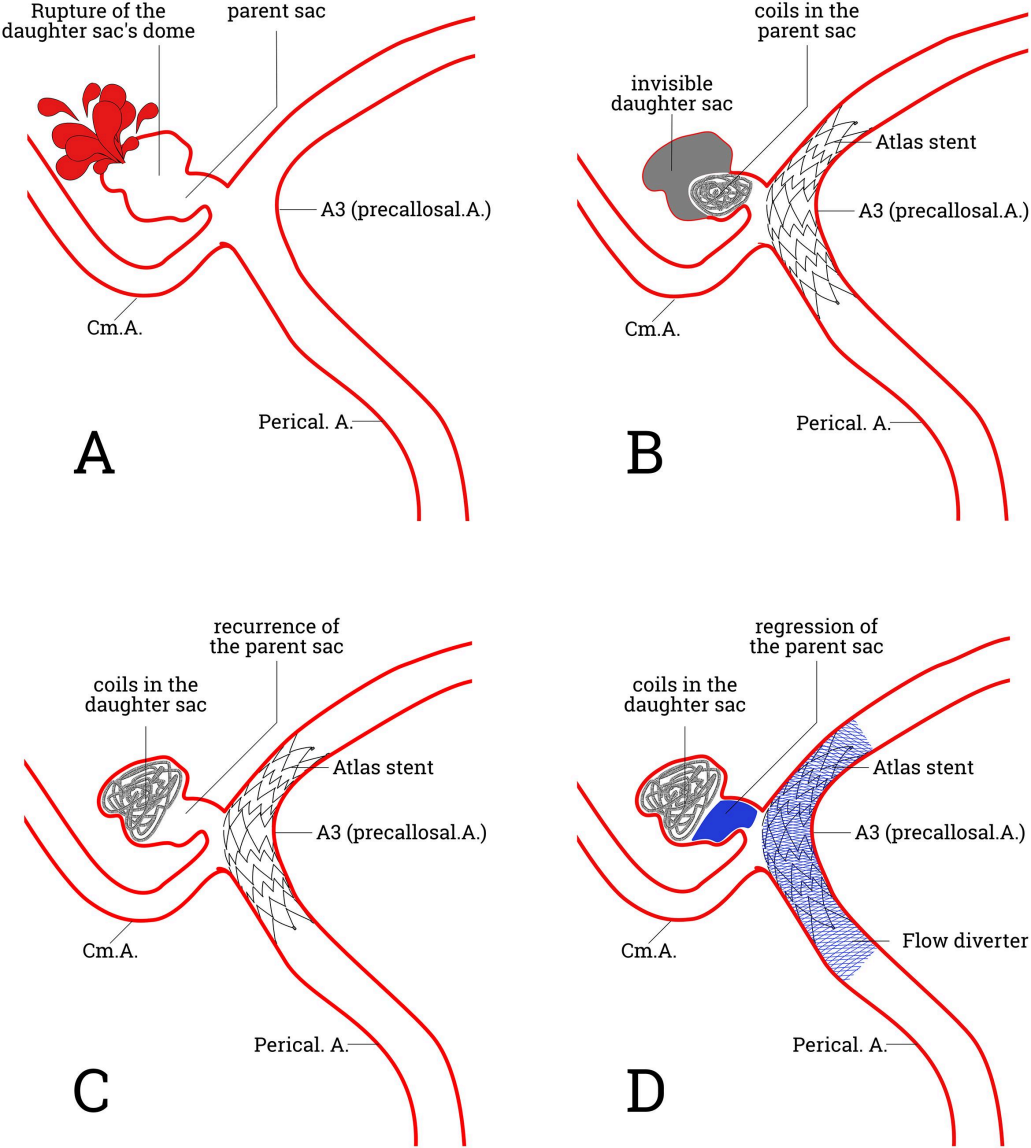
This hypothetical schematic illustrates a potential mechanism for short-term recurrence after the initial Atlas^TM^ stent-assisted colling of a ruptured distal anterior cerebral artery aneurysm: extensive subarachnoid hemorrhage, particularly interhemispheric hematoma, may have obscured the true aneurysm morphology at initial treatment. **A**, The ruptured aneurysm in this case might originally have had an “inverted pear” structure, consisting of a larger distal daughter sac and a smaller proximal parent sac, with the rupture site located at the apex of the daughter sac. **B**, Reduced anterior cerebral artery flow from subarachnoid hemorrhage induced vasospasm and dual-catheter use, coupled with direct hematoma compression of the larger distal daughter sac, prevented its visualization. Consequently, the smaller proximal parent sac was misidentified as the treatment target on preoperative imaging and intraoperatively. **C**, One month follow-up after hematoma resolution and had vasospasm improvement showed the “inverted pear” aneurysm with coils migration into the distal daughter sac, resulting in significant recurrence. **D**, A salvage procedure was performed using a flow diverter, which was telescoped bidirectionally inside the Atlas^TM^ stent, and the aneurysm was successfully treated.

In this case, the ruptured aneurysm may actually present as an “inverted pear” structure (Figure 3A). The massive subarachnoid hemorrhage following the aneurysm rupture led to spasm of the DACA, reducing blood flow on the affected side. Additionally, intraoperative dual catheters further obstructed DACA blood flow, resulting in significant blood flow stagnation within the aneurysm region, particularly in the daughter sac (5). Concurrently, direct external compression from the interhemispheric hematoma obscured the larger distal daughter sac, leading preoperative imaging and intraoperative assessment to misidentify the smaller proximal parent sac as the rupture point (Figure 3B). One month later, the hematoma resolution and improved vasospasm revealed the “inverted pear” aneurysm, with coils from the proximal parent sac migrating into the distal daughter sac, resulting in imaging findings suggestive of significant recurrence (Figure 3C). DACA aneurysms have not been previously reported in the literature as blood blister aneurysms (6), making the likelihood of short-term progression following pseudoaneurysm embolization in this case unlikely. It should be noted that the proposed “inverted pear shaped” structure, though plausible, lacks direct imaging confirmation and thus remains speculative. Further investigation using higher-resolution imaging or intraoperative observation is warranted to validate this finding.

In this DACA case, the microcatheter navigation was unstable and there was a stent in the vessel. Neither traversing the Atlas^TM^ stent mesh nor performing craniotomy for clipping were ideal remedial options. FD reshape blood flow through high metal coverage and low porosity, achieving aneurysm occlusion rates of 50%–100% (7). For recurrent aneurysms after stent-assisted coiling, FD remedial occlusion rates range from 38% to 65% (8). However, the literature primarily focuses on cases near the Willis circle, with technical challenges including the microguidewire “in-out-in” phenomenon, reduced FD visibility due to existing stents or coils, stent interference with new device apposition, and high thromboembolic complication rates (8, 9). No reports exist on FD remedial treatment for recurrent DACA aneurysms after stent-assisted coiling.

From a technical perspective, this DACA case highlighted some key operational points. A 5F intermediate catheter ensured high positioning and coaxiality. Its stable proximal support allowed for continuous microwire rotation and tension-free advancement, with multi-angle projections visualizing significant tip deflection. This confirmed smooth passage through the existing stent lumen to the distal vessel, therapy helping to avoid the “in-out-in” phenomenon. The FD was deployed *in situ* under appropriate tension to avoid dragging the existing Atlas^TM^ stent, covering both ends of the original stent to prevent “endoleak” (8) (Figure 3D).

The long-term occlusion rate for unruptured DACA treated with FD is 82.7% (10), with a branch stenosis rate of 23.8% after covering the callosomarginal artery and a symptomatic ischemic risk of only 3% (11). The branch patency in this case was attributed to smooth stent deployment, adequate dual antiplatelet therapy, and intraoperative tirofiban infusion. The selected Qilin™ FD stent features an optimized braided design, similar to low-profile devices such as the Silk Vista Baby, or the FRED Jr, among others (12–14), which enhances navigability and reduces complication risks.

This case demonstrates that FD remedial treatment is safe and feasible for recurrent aneurysm located on distal (<2 mm) parent arteries with prior conventional stent placement. Given that only 1 month imaging follow-up is currently available, continued long-term observation is warranted to better assess branch vessel patency and aneurysm recurrence. Future efforts should focus on developing FDs specifically designed for small vessels that offer better navigability, enhanced branch vessel protection, and higher metal coverage. Additionally, careful case selection and optimized perioperative antiplatelet management remain essential. Currently, for ruptured DACA aneurysms with high-risk recurrence factors, including unfavorable anatomical morphology, particularly extensive subarachnoid hemorrhage or interhemispheric hematoma, primary standalone coiling is recommended as the first-line option, followed by conventional stent-assisted coiling if necessary, to facilitate potential future FD placement. If risks of DAPT and acute-phase thrombosis are deemed acceptable, direct FD deployment may be an alternative.

## Data Availability

The raw data supporting the conclusions of this article will be made available by the authors, without undue reservation.
